# Vertebral Osteomyelitis Secondary to Bacillus Calmette-Guérin Instillation Therapy for Transitional Cell Carcinoma In Situ

**DOI:** 10.7759/cureus.14589

**Published:** 2021-04-20

**Authors:** Tariq M Jaber, Mohammad Samiullah, Amy Surti

**Affiliations:** 1 Infectious Diseases, Allegheny Health Network, Pittsburgh, USA; 2 Internal Medicine, Broward Health General, Fort Lauderdale, USA; 3 Critical Care Medicine, University of Massachusetts Medical School-Baystate, Springfield, USA

**Keywords:** vertebral osteomyelitis, bcg, mycobacterium bovis, intravesical bcg treatment, bladder cancer

## Abstract

Transitional cell carcinoma is the sixth most common cancer among men and the 17th most common cancer in women. The treatment methods for the condition range from noninvasive chemotherapy to more invasive procedures like cystectomy and complete transurethral resection of bladder tumor (TURBT) followed by intravesical Bacillus Calmette-Guérin (BCG) therapy (BCG). Intravesical BCG treatment is known to be effective as it is associated with increased survivability and long-term benefits, especially in early-stage, minimally-invasive disease. It is generally considered to be safe, even though some adverse reactions have been described. Vertebral osteomyelitis secondary to intravesical BCG therapy is a rare complication but one that has been reported in the literature. Although our patient had multiple comorbidities, including a previous vertebral compression fracture prior to treatment, complications from intravesical BCG treatment should always be considered in the differential. Further multi-center retrospective studies are needed to better ascertain its true risk given its increasing use as a treatment modality for transitional cell carcinoma.

## Introduction

Malignancy of the bladder is one of the most common types of cancer affecting the genitourinary system. Overall, bladder cancer is the sixth most common cancer in men and the 17th most common cancer in women [1]. Treatment modality is selected based on existing risk factors: concurrent comorbidities, functional status, and the stage of tumor progression at diagnosis. Treatment modalities include transurethral resection of visible bladder tumor (TURBT), intravesical Bacillus Calmette-Guérin (BCG) therapy, fulguration therapy, chemotherapy, and cystectomy [2].

Microbial products for the treatment of cancer have been used since it was first proposed in the late 1800s by Dr. William B. Coley, and the idea of using Mycobacteria in the oncology context originated with Raymond Pearl, who observed decreased incidence of cancer lesions in patients with active tuberculosis [3]. BCG therapy as such was successfully applied for the first time in the late 1970s, and its use as a treatment modality for bladder cancer has substantially increased to a point where it has now become the standard of care [2-7]. Intravesical BCG therapy has been associated with decreased progression and risk of recurrence of the disease and compares favorably with intravesical chemotherapy in this respect [3]. Local and systemic complications have indeed been associated with it, although rarely, including (in descending order of prevalence) fever, hematuria, granulomatous prostatitis, pneumonitis and hepatitis, arthralgia, rash, ureteral obstruction, epididymitis, contracted bladder, renal abscess, sepsis, and cytopenia [7]. Other toxic complications have also been reported, such as spondylitis, mycotic aortic aneurysm, spondylodiscitis, septic joints, sepsis, and osteomyelitis [4,7-10]. Mackel et. al have postulated that trauma to the bladder endothelium could serve as a risk factor for the more toxic complications of intravesical BCG therapy [8]. Trauma, for the purposes of their review, included biopsy or resection of the bladder, radical cystoprostatectomy, endovascular aneurysm repair of the abdominal aorta, or motor vehicle accident, while the remaining cases were attributed to instillation of treatment [8]. No identifiable risk factor, however, could be ascertained as predisposing to serious BCG infection [11]. In this report, we describe a case of vertebral osteomyelitis induced by BCG intravesical therapy, which was confirmed by polymerase chain reaction (PCR).

## Case presentation

**Figure 1:**
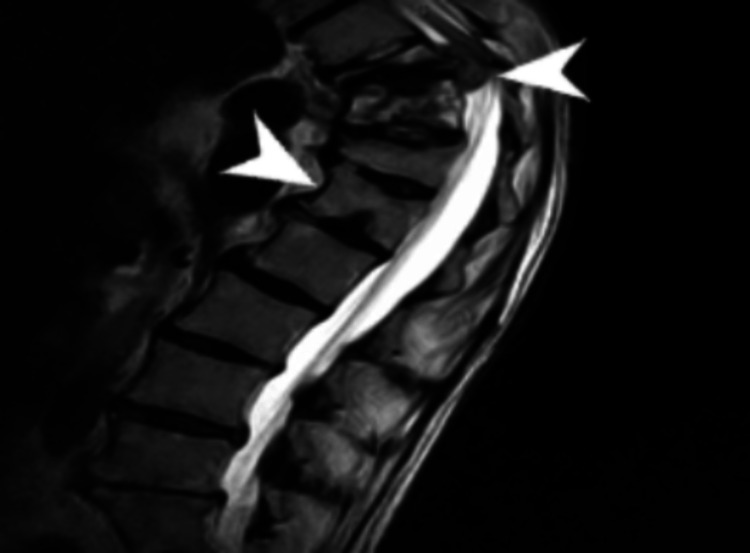
MRI showing acute compression fractures of T10 and T11 Acute compression fractures of T10 and T11 with kyphotic deformity can be seen: 90% height loss at T10, 30% loss at T11. There is also some chronic compression deformity of T12. There is a ventral epidural mass behind the T10 vertebral body causing severe spinal stenosis and compression of the traversing spinal cord MRI: magnetic resonance imaging

We present the case of an 86-year-old male with a known past medical history of transitional cell carcinoma in situ status post multiple fulgurations and intravesical BCG therapy now in remission, osteoporosis, untreated thoracic vertebral compression fractures, and a lower extremity deep vein thrombosis with an inferior vena cava (IVC) filter. The patient complained of a two-week history of multiple falls with an increase in lower extremity numbness, tingling, and gradual weakness. He had undergone an MRI one week prior to the presentation, which had demonstrated a thoracic compression fracture with spinal cord compression (Figure 1). The compression fracture had resulted in worsening symptoms during the following week until he could not ambulate or stand without support but without any fecal and/or urinary incontinence. The patient denied fevers, chills, night sweats, hematuria, or any noticeable symptoms post-BCG therapy. He denied any history of radiation, chemotherapy, or other surgical intervention including TURBT or cystectomy, and was in remission at the time of presentation. His only recent intervention had been fulguration therapy, which had been performed at the time of his last intravesical BCG therapy six months prior to the presentation. The actual number of BCG treatments that he had undergone was not provided by the patient.

Physical exam revealed diminished lower extremity muscle strength (1/5 hip flexion, 3/5 bilateral knee extension, 2/5 ankle plantarflexion, and dorsiflexion). He had a spastic tone and diminished sensation to touch in the lower extremities. Both patellar deep tendon reflexes were hyperactive relative to those of the upper extremities. A sphincter tone was present. The patient also experienced urinary incontinence.

**Figure 2:**
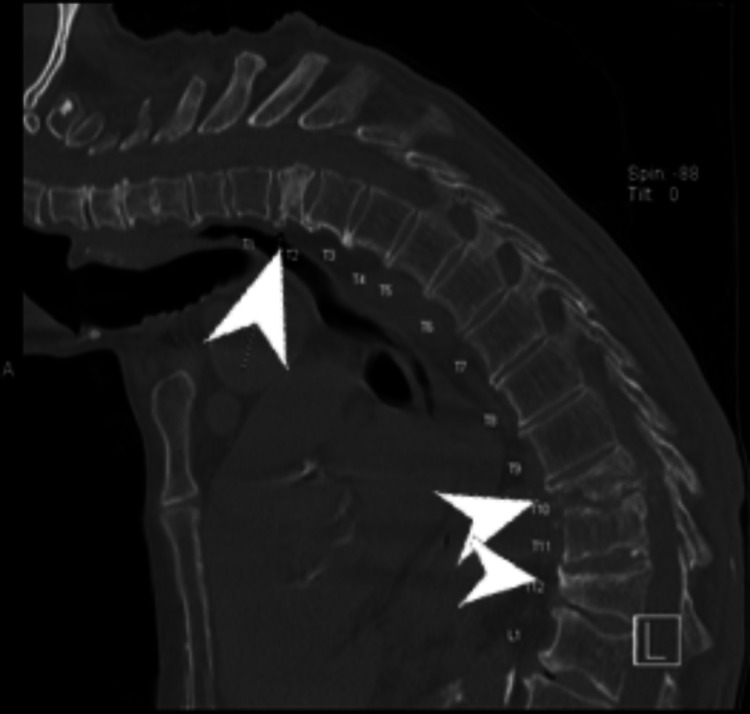
Repeat CT scan of the spine without contrast The image shows severe kyphosis of the thoracic spine. There is a compression fracture of the T2 vertebral body with sclerosis and this is mild compression deformity. There are also severe compression deformities of the T10 and T12 levels with extensive endplate changes in the region CT: computed tomography

**Figure 3:**
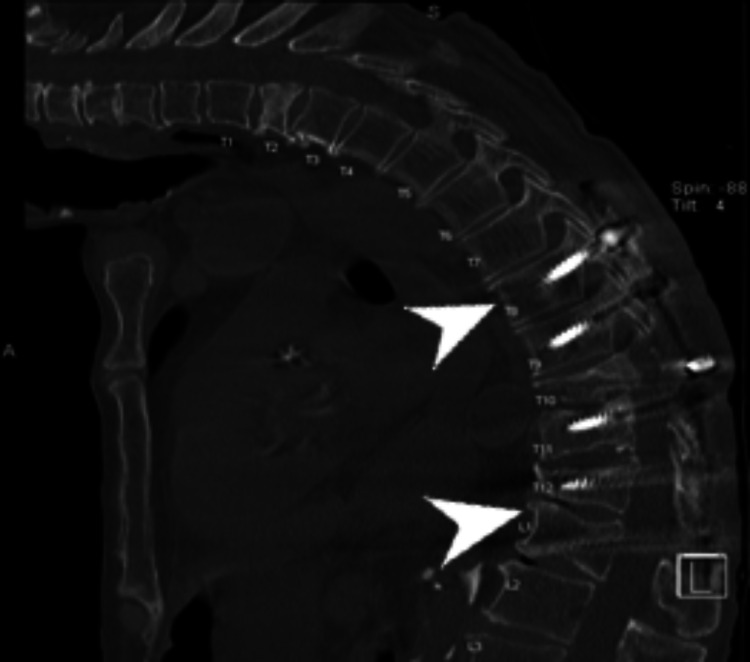
Decompressive thoracic laminectomy The image shows T8-L1 posterior thoracic fusion with laminectomy

Initial labs did not demonstrate leukocytosis, although erythrocyte sedimentation rate (ESR) and C-​reactive protein (CRP) were elevated at 55 mm/hour and 7.69 mg/dL, respectively. Interferon-gamma-release assay and HIV were negative as well. CT imaging without contrast showed severe kyphosis of the thoracic spine in addition to a compression fracture of the T2 vertebral body with destructive lytic lesions with compression deformities of T10 to T12 with extensive endplate changes in the region surrounded by edema (Figure 2). The patient underwent an emergent decompressive thoracic laminectomy (T9-T11) in addition to the resection of a ventral epidural mass of T10 to T11 (Figure 3).

**Figure 4:**
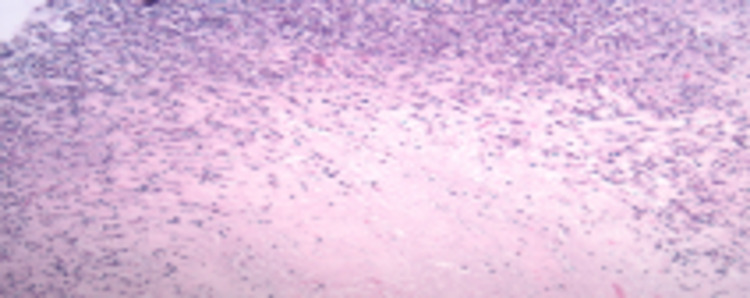
Necrotizing granulomatous inflammation Necrotizing granulomatous inflammation with acid-fast organism. Sampling consists of fibroconnective tissue with necrotizing granulomatous inflammation. A rare possible acid-fast organism is seen with AFB stain. PAS and GMS stains are negative for fungal organisms. Cytokeratin stain is negative for carcinoma AFB: acid-fast bacilli; PAS: periodic acid-Schiff; GMS: Grocott methenamine-silver

The surgical pathology report noted necrotizing granulomatous inflammation with a possible acid-fast organism as seen with acid-fast bacilli (AFB) stain (Figure 4). Periodic acid-Schiff (PAS) and Grocott methenamine-silver (GMS) stains were negative for fungal organisms. Cytokeratin stain was negative for any carcinoma. The pathology report was positive for *Mycobacterium tuberculosis (M. tuberculosis)* complex (16 rRNA), but the Florida State Lab confirmed *Mycobacterium bovis (M. bovis)*. A nine-month treatment course was recommended with the first two months consisting of rifampin, ethambutol, and isoniazid, followed by seven months of rifampin and isoniazid. The patient is currently being followed up on an outpatient basis and has reported post-interventional symptomatic improvements.

## Discussion

*M. bovis* is primarily a zoonotic pathogen found in cattle and other livestock and domesticated animals [12]. The potential for the use of microbial products for the treatment of cancer was first noted in the late 1800s and the utilization of intravesical BCG was initiated with its first successful use for treatment in 1976 [3]. Intravesical BCG therapy is now the standard of care for bladder cancer, especially in the post-TURBT setting [2-10]. The proposed mechanism of action for the attachment of live BCG proceeds via internalization into the abnormally proliferating transitional cells lining the bladder, causing an upregulation of gene expression, which leads to an increase in proinflammatory cytokine factors [3]. Inevitable recruitment of host immune defense follows, involving direct cytotoxicity (NK and CD8 T-Cells) to the cancerous cells [3]. Osteomyelitis is a rare but serious systemic complication of intravesical BCG therapy [2-11]. Other deleterious systemic complications include spondylodiscitis, mycotic aortic aneurysm, and septic arthritis [7-10]. In addition, pulmonary tuberculosis, lymphocytic meningitis, arthritis, and tubulointerstitial nephritis have also been reported [11]. It is thought that *M. bovis* spreads via Batson’s paravertebral venous plexus or translocational hematogenous dissemination, leading to paraplegia [4,13].

One of the more challenging aspects of BCG-related complications is identifying *M. bovis* as the source of the underlying infection given its vague presentation, rarity, low pathogenicity, and incidence in difficult-to-access tissues [10-11]. Differentiating BCG infection by *M. bovis* from that caused by *M. tuberculosis* also presents many challenges. Culturing the organism is itself challenging, and not all laboratories may have the resources to perform such a specific analysis [4]. PCR and other molecular assays are often critical for the diagnosis [4,10].

An important risk factor is trauma to the urothelial tissue [8-9,11,14]. Trauma could manifest as a result of TURBT, pre-existing urinary tract infection (UTI), bladder catheterization, aseptic cystitis, indeed any comorbid condition that could cause hematuria [14]. However, in a single-center retrospective study, an obvious risk factor or predictor for the development of BCG-related infection post-treatment could not be identified [11]. That review incorporated timing of post-transurethral surgery, number of prior BCG instillations, comorbidities, history of tuberculous, and immunosuppression [11]. As per the American Urological Association, current contraindications to BCG instillation include bladder and/or prostatic surgery including biopsy within 7-14 days of BCG instillation [15]. Also included are traumatic catheterizations or gross hematuria on the day of treatment [15]. Other contradictions include pregnancy and/or lactating patients, active tuberculosis, immunosuppression, cancer therapy, immunosuppressive therapy, symptomatic UTI, presence of fever, any use of antibiotics that interfere with the effectiveness of BCG modality of treatment, and allergies to BCG itself [15].

Despite its effectiveness, BCG therapy is costly and often comes with risks [14]. The optimal BCG dose remains a controversial subject [14,16]. It has been demonstrated in vitro that the BCG dose has a direct impact on cellular response and relative anti-tumor effect [17]. To this end, it may be advantageous to incorporate higher doses. However, higher doses are associated with an increased risk of complications, and hence providing an optimal dose while reducing morbidity remains the subject of investigation [18]. In an efficacy analysis, there was a slight increase in overall survivability with low-dose BCG compared to full-dose treatment, but with an increased risk of relapse [14]. Even the optimal maintenance dose post-induction has yet to be fully determined [16]. 

The only notable risk factor for our patient, aside from his age, was the recent fulguration therapy. Fulguration therapy for urothelial tumors is considered low risk for bladder wall damage as it delivers heat current to a targeted tissue [19]. It minimizes uroepithelial lining breaks that occur with surgical excision and does not pose any immunosuppressive effects, unlike chemotherapy. Thus, fulguration therapy, while damaging, created the necessary conditions that induced hematogenous spread of *M. bovis* from the BCG vaccine despite the lack of symptoms. The patient was not immunocompromised, nor had he received prior radiation, chemotherapy, or TURBT procedure. He denied any local and systemic manifestations of post-BCG therapy (e.g., fever, night sweats, frequency, urgency, and especially hematuria). He did not present any signs of active infection even during his initial presentation at our hospital, aside from elevated ESR and CRP. His initial presentation was unique given his history of compression fracture, and osteoporosis, which may have contributed to the development of osteomyelitis by *M. bovis*.

This article was presented in the abstract form at the American College of Physicians National Conference in Philadelphia.

## Conclusions

*M. bovis* osteomyelitis is a rare complication of intravesical BCG therapy. Although our patient had multiple comorbidities, including a previous vertebral compression fracture prior to treatment, complications from intravesical BCG treatment should always be considered in the differential. Further multi-center retrospective studies are needed to better understand the true risks associated with the therapy, especially given its increasing use as a treatment modality for transitional cell carcinoma.
